# Uterine inflammatory myofibroblastic tumor: a retrospective analysis

**DOI:** 10.3389/fonc.2024.1461092

**Published:** 2024-09-20

**Authors:** Liping Bai, Ling Han, Ai Zheng, Yali Chen

**Affiliations:** ^1^ Department of Obstetrics and Gynecology, West China Second University Hospital, Sichuan University, Chengdu, China; ^2^ Key Laboratory of Birth defects and Related Diseases of Women and Children (Sichuan University), Ministry of Education, Chengdu, China

**Keywords:** inflammatory myofibroblastic tumor, anaplastic lymphoma kinase, uterine leiomyoma, diagnosis, uterus, retrospective analysis

## Abstract

**Objective:**

Uterine inflammatory myofibroblastic tumor (UIMT) is a rare tumor of the female reproductive tract with uncertain malignant potential. Previous case series reports have limited our understanding of its diagnosis and treatment. Therefore, we conducted a retrospective analysis of patient files at West China Second University Hospital, Sichuan University to contribute valuable clinical insights to future treatment strategies for this disease.

**Method:**

We comprehensively reviewed patient files of individuals diagnosed with UIMT from January 1st, 2013 to May 1st, 2023.

**Results:**

We included twenty-seven cases of uterine inflammatory myofibroblastic tumor in our study. Of these, 51.85% (14 cases) were diagnosed with abnormal uterine bleeding, 2 cases had dysmenorrhea, and 12 were unexpectedly diagnosed with suspected uterine fibroids. Ten cases performed total hysterectomy, and 17 cases underwent lesion resection. The positive rate of anaplastic lymphoma kinase (ALK) immunohistochemistry reached 96.3%. After a median of 8 months follow-up time, all patients were disease-free and had survived.

**Conclusion:**

Uterine inflammatory myofibroblastic tumor is easily misdiagnosed, making its diagnosis challenging. Histological features, immunohistochemical results, and molecular confirmation using fluorescence *in situ* hybridization (FISH) or Next-generation sequencing should be used to confirm the diagnosis. Positive ALK immunohistochemistry, ALK rearrangement, ALK fusion are helpful in diagnosis and ALK inhibitor therapy. Total hysterectomy is often performed for women who do not require fertility, while lesion resection and close follow-up may be considered for those who require fertility preservation.

## Introduction

Inflammatory myofibroblastic tumor (IMT), a rare condition characterized by the presence of fibroblastic spindle cells with varying degrees of myxoid stroma and lymphoplasmacytic inflammation (1). Although initially discovered in the lungs, IMT can occur in various anatomical locations, including the female reproductive organs, particularly the uterus. In the uterus, IMT is responsible for only 0.1% of "leiomyomas", but its incidence rises to 10% in pregnant women and 14% in uterine smooth muscle tumors of uncertain malignant potential(STUMP) (2). Most cases of uterine inflammatory myofibroblastic tumor (UIMT) are benign, but the tumor can spread to surrounding tissues or recur after surgery. IMT often involves ALK gene rearrangements and has a low risk of metastasis. It is classified as a tumor of intermediate malignant potential, with recurrence and metastasis rates of approximately 25% and 2%, respectively (3).

Diagnosing IMT can be challenging due to its similarity to other conditions such as leiomyomas, leiomyosarcomas, endometrial stromal tumors, fibroleiomyomatosis, and ligamentous fibroma. The clinical overlap in morphology often leads to misdiagnosis, potentially resulting in a higher incidence than originally estimated. However, advancements in diagnostic tools, such as ALK immunohistochemical testing, have improved the ability to differentiate between STUMP and leiomyosarcoma. In fact, ALK immunohistochemistry, FISH, and RNA sequencing have proven to be highly specific in diagnosing UIMT, enabling its diagnosis with any positive result. Some ALK-negative cases can have other gene abnormalities, including RET, ROS1, or NTRK3 fusion, as well as PDGFRB 3’ fusion (4), THBS1, IGFBP5, DES, SEC31, TPM3, and TIMP3. DES-ALK, THBS1, FN1, DCTN1, and PPP1CB (5, 6). Additionally, certain fusion genes, such as TIMP3-ALK and THBS1-ALK, have been found to be highly enriched in pregnancy-related UIMT (7). However, the current diagnosis still relies on histological examination combined with immunohistochemistry, with ALK protein expression and ALK gene rearrangement serving as strong evidence for IMT diagnosis.

Currently, there is a lack of guidelines for the diagnosis and treatment of UIMT. Due to the rarity of the disease, previous literature has primarily consisted of individual case reports. Further research is needed to develop diagnostic and treatment methods for UIMT. The objective of this article is to conduct a retrospective analysis of UIMT patients in our hospital and contribute valuable clinical insights to future treatment strategies for this disease.

## Materials and methods

### Study population and data sources

This retrospective, observational, single-center study was conducted at West China Second University Hospital, Sichuan University, Chengdu, China, after receiving ethical approval from the hospital’s Ethics Committee. Written informed consent was obtained from all 27 patients who were diagnosed with uterine inflammatory myofibroblastic tumor and underwent surgery at the hospital between January 1, 2013, and May 11, 2023.

The study retrospectively examined patients’ clinical and pathological information. The pathological specimen was independently reviewed by two pathologists from West China Second University Hospital. Basic patient information, including age, symptoms, tumor characteristics (such as location and size), surgical procedures, comorbidities, adjuvant therapy, recurrence and metastasis rates, follow-up duration, and pregnancy status, was collected. Pathological features, including ALK immunohistochemistry, ALK fluorescence *in situ* hybridization, FH, CD10, caldesmon, desmin, Ki67, border, growth pattern, cell type, nuclear atypia, mitotic index, necrosis, lymphovascular invasion, type and extent of inflammatory infiltrates, as well as primary or metastatic status, were also collected. Oncological outcomes were assessed by following up with patients through outpatient visits and phone calls. Currently, all patients are disease-free, and no recurrence or metastasis has been reported.

### Statistical analysis

For continuous normally distributed variables, mean ± standard deviation was used, and the t-test was used for analysis. The Levene’s test assessed variance homogeneity. For non-normally distributed continuous variables, medians (range) were used and analyzed using the Wilcoxon-Mann-Whitney U test. Pearson’s χ2 test or Fisher’s exact test was used for categorical variables. The likelihood ratio test was used to compare groups of categorical variables. SPSS version 25.0 (IBM Corp, Armonk, NY, USA) was used for statistical analysis. A two-sided p-value <0.05 was considered statistically significant. Descriptive analysis was used for non-continuous variables, and when the p-value was <0.05, the median and interquartile range were used.

## Results

### Clinical features

The study included 27 patients who were pathologically diagnosed with uterine inflammatory myofibroblastic tumor (UIMT). The patients' basic characteristics are presented in **Tables 1**–**3**. UIMTs have been documented in patients aged 21 to 64 years, with a median age of 42 years, and the tumors varied in size from 3 to 8 cm, with a median diameter of 5 cm. Abnormal uterine bleeding was the most common symptom, occurring in 51.85% of cases. Two cases had dysmenorrhea, and 12 cases were incidentally diagnosed due to suspected uterine fibroids. Notably, patient 19 had both dysmenorrhea and abnormal uterine bleeding, patient 13 was unexpectedly diagnosed during a cesarean section, and patient 23 during the removal of an ovarian cyst. The majority (21) of cases were located within the intramuscular layers of the uterus, while 3 were subserosal and 3 were submucosal. All cases of IMT occurred in the uterine body without any extrauterine lesions.

**Table 1:** Clinical characteristics of uterine IMT.

Case	Age	Symptoms	Location	Tumor size(cm)	Surgical procedure	Adjuvant therapy	Recurrence/metastasis	Follow up time(months)	pregnancy	status
1	46	AUB	IUC	5	HYS	No	No	4	No	NED
2	50	AUB	IUC	6	HYS	No	No	3	No	NED
3	64	PLM	IUC	6	HYS	No	No	7	No	NED
4	57	AUB	IUC	6	HYS	No	No	7	No	NED
5	46	AUB	IUC	5	HYS+BSO+PLA	chemotherapy	No	4	No	NED
6	50	PLM	IUC	6	HYS	No	No	10	No	NED
7	49	PLM	IUC	5.5	HYS	No	No	3	No	NED
8	43	AUB	SMUC	7	HYS	No	No	8	No	NED
9	43	PLM	IUC	4	MYO	No	No	4	No	NED
10	44	DYS	SMUC	5	MYO	No	No	50	No	NED
11	33	AUB	SMUC	4	MYO	No	No	2	No	NED
12	34	PLM	IUC	6	MYO	No	No	6	No	NED
13	32	Other symptoms*	IUC	3	MYO	No	No	6	No	NED
14	46	AUB	IUC	8	MYO	No	No	7	No	NED
15	32	PLM	IUC	5	MYO	No	No	7	No	NED
16	32	PLM	SSUC	6	MYO	No	No	11	No	NED
17	30	PLM	IUC	6	MYO	No	No	17	No	NED
18	39	AUB	IUC	5	MYO	No	No	29	No	NED
19	45	DYS+ AUB	IUC	3	MYO	No	No	20	No	NED
20	27	PLM	IUC	4	MYO	No	No	9	No	NED
21	32	AUB	IUC	3	HYS	No	No	19	No	NED
22	42	AUB	IUC	3.8	HYS	No	No	31	No	NED
23	29	Other symptoms#	SSUC	3	MYO	No	No	9	No	NED
24	30	AUB	IUC	3.8	MYO	No	No	45	Yes	NED
25	30	PLM	IUC	5	MYO	No	No	15	No	NED
26	46	AUB	IUC	6	MYO	No	No	6	No	NED
27	21	AUB	SMUC	4	MYO	No	No	18	No	NED

AUB, abnormal uterine bleeding; NED, no evidence of disease; IUC, intramural of uterus corpus; SMUC, Submucosa of uterus corpus; SSUC, Subserosal of uterus corpus; BSO, Bilateral salpingo-oophorectomy; HYS, hysterectomy; MYO, myomectomy; PLA, pelvic lymphadenectomy; PLM, presumed leiomyoma; DYS, dysmenorrhea.

Other symptoms*: Detected during cesarean section.

Other symptoms#: Detected during oophorocystectomy.

**Table 2:** The pathological features of the patient.

case	ALK IHC	ALK FISH	caldesmon	FH	CD10	DESMIN	Ki67	border	growth pattern	nuclear atypia	mitotic index(per 10 HPFs)	necrosis	LVSI	type and extent of inflammatory infiltrate
1	P	N	P	P	N		5	well-circumscribed	hyalinized	mild	5%	No	No	patch
2	P	ND	P	P	N		<5	well-circumscribed	hyalinized	mild	10%	No	No	patch
3	N	P		P	P		1	well-circumscribed	myxoid	mild	10%	No	No	patch
4	P	P	P	P	N	P	<3	well-circumscribed	compact fascicular	mild	3%	No	No	patch
5	P	ND	P		P	P	40	infiltrative	myxoid	moderate	40	No	yes	diffuse
6	P	P	P	P	N		3	well-circumscribed	hyalinized	moderate	3%	No	No	diffuse
7	P	ND	P	P	N		<5	well-circumscribed	hyalinized	moderate	5%	No	No	patch
8	P	P	N	P	P	P	20	infiltrative	myxoid	moderate	20	yes	No	diffuse
9	P	P	P	P	N		<5	well-circumscribed	hyalinized	mild	5%	No	No	patch
10	P	ND	N		N	P	30	well-circumscribed	hyalinized	moderate	30%	No	No	diffuse
11	P	P		P	N		5-10	well-circumscribed	compact fascicular	moderate	10%	No	No	diffuse
12	P	P		P	N		5-10	well-circumscribed	compact fascicular	moderate	10%	No	No	diffuse
13	P	ND	P	P	P	P	3	well-circumscribed	compact fascicular	moderate	3%	No	No	patch
14	P	N	P	P	P		5	well-circumscribed	myxoid	moderate	3%	No	No	diffuse
15	P	P		P	N		1	well-circumscribed	hyalinized	mild	1%	No	No	patch
16	P	P		P	N		3-5	well-circumscribed	hyalinized	mild	5%	No	No	patch
17	P	P	P	P	N		<5	well-circumscribed	hyalinized	moderate	5%	No	No	diffuse
18	P	ND	P		N		<1	well-circumscribed	hyalinized	moderate	1%	No	No	diffuse
19	P	P	P	P	N		5	well-circumscribed	myxoid	moderate	5%	No	No	diffuse
20	P	P	P	P	P	P	<3	well-circumscribed	compact fascicular	moderate	3%	No	No	diffuse
21	P	P	P	P	N		5-10	well-circumscribed	myxoid	moderate	10%	No	No	diffuse
22	P	ND	P		N	P	5	well-circumscribed	myxoid	moderate	5%	No	No	diffuse
23	P	P	P	P	N		<5	well-circumscribed	compact fascicular	mild	5%	No	No	patch
24	P	ND	P		N		3-5	well-circumscribed	hyalinized	moderate	5%	No	No	diffuse
25	P	P	P	P	P		<5	well-circumscribed	hyalinized	moderate	5%	No	No	diffuse
26	P	ND		P	N		<5	well-circumscribed	hyalinized	mild	5%	No	No	diffuse
27	P	P	P	P	N		5-10	well-circumscribed	myxoid	mild	10%	No	No	diffuse

LVSI, Lymph vascular space invasion; P, positive; N, negative; ND, not done.

In the cell type detection, except for case No. 23, where the majority was spindled and focal was epithelioid, all the other cases were spindled.

**Table 3:** Clinical and pathological characteristics of the patients.

Parameter	
Age(years)	
Range	21-64
Median	42
Mean	39.7 ± 9.9
Location	
Corpus	27
Cervix	0
Symptom	
AUB	14(51.85%)
dysmenorrhea	2(7.41%)
Incidental finding	12(44.44%)
Size	
Range	3-8
Median	5
Mean ± SD	5 ± 1.35
Surgical procedure	
Tumor resection	17(62.96%)
HYS	10(37.04%)
Follow up months	
Range	2-50
Median	8
Mean ± SD	13 ± 12.2
Pregnancy	
Yes	1(5.88%)
No	16(94.12%)
Borders	
well-circumscribed	25(92.59%)
infiltrative	2(7.41%)
ALK IHC	
Positive	26(96.30%)
Negative	1(3.7%)
ALK FISH	
Rearragement	16(59.26%)
Normal	2(7.41%)
Not done	9(33.33%)
Mitoses	
<5/10 HPF	7(25.93%)
≥5/10 HPF	20(74.07)
Atypia	
No/Mild	10(37.04%)
Moderate/Severe	17(62.96%)
Necrosis	
Yes	1(3.7%)
No	26(96.3)
Inflammation	
Diffuse	17(62.96%)
Patch	10(37.04%)
LVSI	
Yes	1(3.7%)
No	26(96.3%)

### Pathological and molecular results

**Figure 1:**
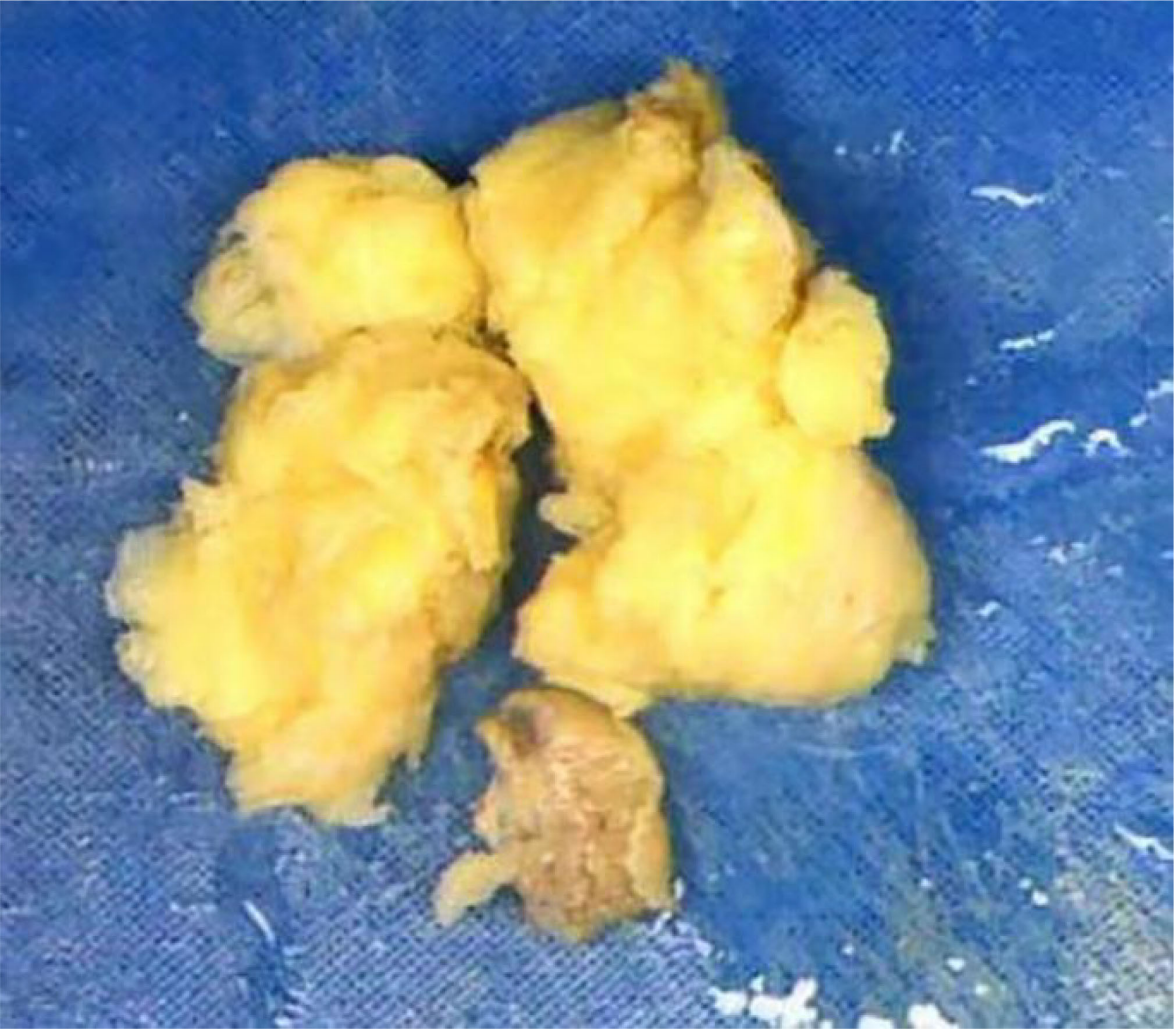
Uterine inflammatory myofibroblastic tumor. Macroscopically, most tumors were tan, pink, or white. The sectioned surface of the tumor can be of soft consistency, with a whorling appearance, hemorrhage, necrosis.

**Figure 2:**
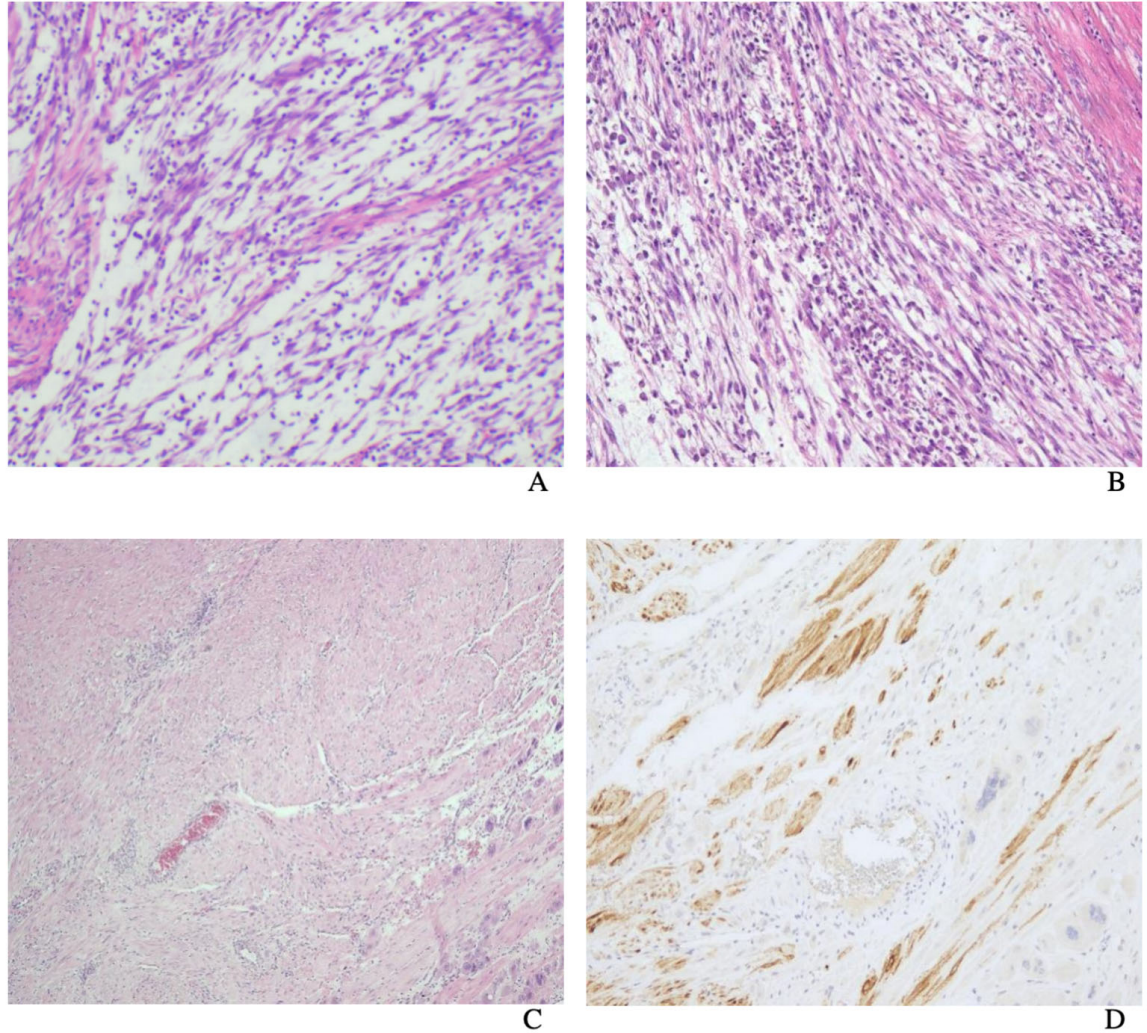
Uterine inflammatory myofibroblastic tumor. The tumor cells are usually fusiform cells dispersed in a myxoid extracellular matrix with varying degrees of inflammatory infiltrates **(A)**. Tumor cells are composed of spindle and epithelioid cells admixed with lymphocytes **(B)**. Uterine inflammatory myofibroblastic tumor. H&E **(C)** shows the appearance of tumor cells mimicked that of a smooth muscle tumor (smooth muscle-like) and ALK immunostain **(D)**.

Macroscopically, most tumors were tan, pink, or white with no apparent capsule, and the sectioned surface of the tumor could have soft consistency, with a whorling appearance, hemorrhage, necrosis, myxoid features, and cyst formation (**Figure 1**). Tumor cells often comprised plump fusiform cells dispersed in a myxoid extracellular matrix with inflammatory infiltrates of varying amounts, while less commonly, the tumor cells may be epithelioid cell-like (**Figure 2**). 10 cases showed patchy infiltration, while 17 cases showed diffuse infiltration, and only 1 case exhibited lymphovascular space invasion (LVSI). Twenty-six cases tested positive for ALK immunohistochemistry, with only one case showing a negative result for ALK immunohistochemistry but a positive result for ALK FISH analysis. ALK FISH analysis was carried out on eighteen cases; sixteen cases showed positive results, two tested negative, and 9 cases did not undergo FISH testing.

### Treatment outcomes

Of the 27 patients, 10 underwent total hysterectomy, and 17 had lesion removal. Patient 5 underwent total hysterectomy, bilateral salpingectomy, and pelvic lymph node dissection, followed by adjuvant chemotherapy due to infiltrative growth, while patient 24 opted for fertility preservation and had a successful natural pregnancy after surgery, culminating in a term cesarean section. During the follow-up period, which ranged from 2 to 50 months, all patients remained disease-free. Additionally, the study found no significant difference in prognosis between tumor excision and hysterectomy, suggesting that alternative treatment options may be effective in managing UIMT.

## Discussion

Our recent analysis of clinicopathological data at our hospital has provided new insights into the nature of uterine inflammatory myofibroblastic tumor (UIMT). The exact cause of IMT remains elusive, but it is believed that potential triggers may include traumatic injuries, bacterial or EB viral infections, chromosomal abnormalities, abnormal repair processes, severe surgeries, and inflammation spreading (8). According to the latest 2020 WHO definition, IMT is a distinctive and rare tumor characterized by a combination of spindle-shaped myofibroblast cells, fibroblast cells, plasma cells, eosinophils, lymphocytes, and other inflammatory cells, with minimal potential for metastasis (9).

Distinguishing UIMT from uterine leiomyoma based on morphology alone can be challenging due to the atypical clinical features commonly seen in UIMT. Recent research has shown that 0.3% of unselected leiomyomas were reclassified as IMTs based on positive anaplastic lymphoma kinase (ALK) immunohistochemistry (IHC) (4), even though routine ALK screening for the diagnosis of leiomyomas is not currently recommended. Our own study found that the median age of UIMT patients was 42 years, in line with previous reports by Karpathiou G. and Shukla PS (2, 10)., who observed a wide age range from 6 to 73 years, with a median age of 39 years. Abnormal uterine (51.85%) and the incidental discovery of uterine fibroids were the most common patient complaints. It is worth noting that many patients were asymptomatic, leading to a high rate of clinical underdiagnosis of UIMT. In other reports, IMT in other parts of the body, such as abdominal IMT, is more common in children and adolescents (11, 12). And the clinical manifestations of these IMTs are nonspecific and broad spectrum, ranging from asymptomatic to severe systemic symptoms (11, 12).

UIMTs are often mistaken for uterine leiomyomas, but they exhibit different texture and morphology, characterized by a softer, gelatinous consistency, and clear or irregular borders. They can be classified histologically into myxoid/vascular type, dense spindle cell type, or hypocellular fibrous type, with varying amounts of chronic inflammatory cells. They express ALK as well as smooth muscle markers (SMA, Desmin, Caldesmon), CD10, and others (6). ALK demonstrates high sensitivity and specificity in diagnosing IMT, with ALK gene rearrangement occurring at the 2p23 locus (13). Previous studies have reported ALK positivity rates of 87.5-100% (4), and in our study, the ALK IHC positivity rate reached 96.3%. Various ALK fusion partners have been identified in the literature on IMT (CARS, TPM4, TPM3 EML4, RANBP2, IGFBP4, ATIC, CLTC, etc.), while ALK-negative cases may show ROS1 and PDFGRB alterations, and a small proportion has ETV6-NTRK3 fusion (14). TIMP3 and THBS1 genes are more commonly identified fusion partners in pregnancy-related IMTs (15). Therefore, if ALK is negative, further RNA sequencing is needed to assist in the diagnosis. Nevertheless, it is crucial to note that ALK positivity is a key factor in diagnosing IMT. It is worth mentioning that no ALK fusion genes have been detected in uterine leiomyomas thus far. The current essential diagnostic criteria for IMT include the presence of spindle cell arrangement, infiltration of lymphocytes and plasma cells, expression of SMA, and frequent expression of ALK or ROS1. Notably, IMTs associated with pregnancy exhibit morphological features such as varying amounts of myxoid stroma and lymphoplasmacytic infiltrates (7).

The primary treatment for women diagnosed with noninvasive UIMT who do not require fertility preservation is total hysterectomy, while those who require fertility preservation may consider lesion resection with close follow-up. This approach is in line with previous studies. Prior to 2014, all reported cases of uterine IMT in the literature had a benign clinical course without evidence of recurrence or metastasis after surgery (1). However, research on UIMT invasiveness has been limited to the past decade. However, in patients with other IMT sites that have been reported, they still have a low recurrence rate after conservative surgery and treatment (11, 12)?.

In our case series, the invasive UIMT had a diameter of 5 cm, infiltrative borders, positive lymphovascular space invasion (LVSI), and 40 mitoses in 10 high-power fields. Malignant IMTs have been reported to have a lesion size ≥10.5 cm, severe nuclear atypia, ≥18 mitoses in 10 high-power fields, and features of lymphovascular invasion (5, 6). Studies on the invasiveness of IMT have found that myxoid dominance transitions to dense/spindle dominance in cases of multiple recurrences (16). Bennett’s study found that the dense/spindle type was associated with recurrence (5), while other studies have found that myxoid dominance is associated with a higher risk (1). The latest classification by the World Health Organization includes characteristics such as tumor diameter >7 cm, moderate to severe cytological atypia, increased mitotic index, necrosis, and lymphovascular infiltration. However, there are also reports of cases of metastasis and recurrence without these features.

Treatment for recurrent or invasive IMT usually involves a combination of chemotherapy, targeted therapy, or surgery. Tumors with larger diameters, typical nuclear atypia, lymphovascular infiltration, high mitotic count, and tumor necrosis are associated with poor outcomes (4). Understanding the genetics of IMT can facilitate targeted therapies. Approximately 10% of uterine IMTs are ALK-negative, and FISH or RNA sequencing is helpful for diagnosis (15). Confirmed ALK positivity has both diagnostic and therapeutic implications. ALK inhibitors, such as crizotinib, have shown effectiveness in treating recurrent and/or refractory invasive diseases (4, 17). Pregnancy appears to have a certain correlation with IMT, and targeted therapy using tyrosine kinase inhibitors may represent a new treatment option (17, 18).

Recent studies have indicated that minimally invasive procedures such as laparoscopy and hysteroscopy are preferred for treating UIMT, while careful monitoring and continued follow-up are effective strategies for women seeking to preserve fertility (17). However, UIMTs are rare tumors, and the understanding mostly comes from case reports with short follow-up periods, which can lead to selection bias in retrospective analyses.

## Conclusion

Uterine inflammatory myofibroblastic tumor (UIMT) is a rare tumor that can be challenging to diagnose and treat. Total hysterectomy is often performed for women who do not require fertility, while lesion resection and close follow-up may be considered for those who require fertility preservation. ALK protein expression and gene rearrangements are used for diagnostic confirmation. Tumors with larger size, nuclear atypia, lymphovascular invasion, high mitotic count, and necrosis are associated with poorer outcomes. Improved understanding of the genetics and invasiveness of IMT is necessary for developing effective treatment strategies.

## Data Availability

The original contributions presented in the study are included in the article/supplementary material. Further inquiries can be directed to the corresponding author.
